# Retraction: Potentiation of the anticancer effects of everolimus using a dual mTORC1/2 inhibitor in hepatocellular carcinoma cells

**DOI:** 10.18632/oncotarget.28910

**Published:** 2026-09-08

**Authors:** Jong-Ok Kim, Kee-Hwan Kim, In Sang Song, Kwang-Sik Cheon, Ok-Hee Kim, Sang Chul Lee, Sang Kuon Lee, Say-June Kim

**Affiliations:** ^1^Department of Pathology, Daejeon St. Mary’s Hospital, College of Medicine, The Catholic University of Korea, Seoul, Republic of Korea; ^2^Department of Surgery, Uijeongbu St. Mary’s Hospital, College of Medicine, The Catholic University of Korea, Seoul, Republic of Korea; ^3^Department of Surgery, Chungnam National University Hospital, Daejeon, Republic of Korea; ^4^Department of Surgery, Daejeon St. Mary’s Hospital, College of Medicine, The Catholic University of Korea, Seoul, Republic of Korea; ^*^These authors contributed equally to this work

**This article has been retracted:** Oncotarget has concluded its investigation of this paper, and the following issues were discovered:

- Figure 1: The beta-actin blots of Figure 1C and 1D, and of 1G and 1H are duplicates, yet, they represent different cell types (HepG2 (C and G) and Hep3B (D and H)).
- Figure 2C: The beta-actin blot was reused in Figure 3A of a 2018 paper [1] of the same group of authors, despite reporting a different treatment.
- Figure 2D: The beta-actin blot is the duplicate of the beta-actin blot in Figure 3C of article from the same lab [2], presenting 17-DMAG treatment of AGS cells.
- Figure 2E: The E-cadherin blot is the duplicate of the N-cadherin blot in Figure 4D of 2015 paper [3] of the same group of authors.

Oncotarget contacted the corresponding author, Dr. Say-June Kim, who stated that “these errors were not intentional. They are understood to have arisen during the assembly and organization of the figures. Despite our best efforts, we have been unable to recover the complete original, uncropped source data with intact time and date stamps for the figures in question. For these reasons, we agree that retraction is the appropriate course of action to correct the scientific record.” The absence of original data and multiple instances of duplication compromise the reliability of the reported findings. Consequently, the editorial decision has been made to retract this article. All authors have agreed with this decision.

Original article: Oncotarget. 2017; 8:2936–2948. 2936-2948. https://doi.org/10.18632/oncotarget.13808
